# First genetic linkage map of *Lathyrus cicera* based on RNA sequencing-derived markers: Key tool for genetic mapping of disease resistance

**DOI:** 10.1038/s41438-018-0047-9

**Published:** 2018-09-01

**Authors:** Carmen Santos, Nuno Felipe Almeida, Mara Lisa Alves, Ralf Horres, Nicolas Krezdorn, Susana Trindade Leitão, Thaïs Aznar-Fernández, Björn Rotter, Peter Winter, Diego Rubiales, Maria Carlota Vaz Patto

**Affiliations:** 1 Instituto de Tecnologia Química e Biológica António Xavier, Universidade Nova de Lisboa, Av. da República, Oeiras, 2780-157 Portugal; 2 GenXPro GmbH, Frankfurt am Main, D-60438 Germany; 3 Institute for Sustainable Agriculture, CSIC, Córdoba, E-14004 Spain

## Abstract

The *Lathyrus cicera* transcriptome was analysed in response to rust (*Uromyces pisi*) infection to develop novel molecular breeding tools with potential for genetic mapping of resistance in this robust orphan legume species. One RNA-seq library each was generated from control and rust-inoculated leaves from two *L. cicera* genotypes with contrasting quantitative resistance, *de novo* assembled into contigs and sequence polymorphisms were identified. *In toto*, 19,224 SNPs differentiate the susceptible from the partially resistant genotype’s transcriptome. In addition, we developed and tested 341 expressed E-SSR markers from the contigs, of which 60.7% varied between the two *L. cicera* genotypes. A first *L. cicera* linkage map was created using part of the developed markers in a RIL population from the cross of the two genotypes. This map contains 307 markers, covered 724.2 cM and is organised in 7 major and 2 minor linkage groups, with an average mapping interval of 2.4 cM. The genic markers also enabled us to compare their position in *L. cicera* map with the physical position of the same markers mapped on *Medicago truncatula* genome, highlighting a high macrosyntenic conservation between both species. This study provides a large new set of genic polymorphic molecular markers with potential for mapping rust resistances. It represents the first step towards genomics-assisted precision breeding in *L. cicera*.

## Legumes: Tools for mapping genes for rust resistance

Genetic analysis of *Lathyrus cicera*, or ‘chickling pea’, a plant most commonly cultivated for animal feed, identified DNA polymorphisms that may be associated to resistance to the fungal disease known as rust. Researchers in Europe led by Maria Carlota Vaz Patto at the NOVA University of Lisbon, Portugal, discovered thousands of genetic differences between plants that were either susceptible to or at least partially resistant to the rust fungus *Uromyces pisi*. They created a ‘genetic linkage map’, which reveals the likelihood that different genetic characteristics will be inherited together through the generations. This map can now serve as a tool to help geneticists and plant breeders understand and promote resistance to rust and potentially other fungal diseases in *L cicera*. The results may also be applied to other legumes, including peas and beans more commonly cultivated for human consumption.

## Introduction

*Lathyrus cicera* L., the chickling pea, is an annual legume belonging to the tribe Fabeae^1,2^. It is mainly grown as feedstock, both as fodder and grain^3^. However, as far as human consumption is concerned, *L. cicera* is eaten uncooked as green snack^4^. *L. cicera* adapts well to harsh environments. It is resistant to drought, waterlogging^5^ and to several important legume pathogens. Sources for resistance to rust^6^, powdery mildew^7^, bacterial blight^8^ and crenate broomrape^9^ have been identified. Thus, *L. cicera* is a good option for cropping systems in marginal lands and can function also as a source of resistance genes for transfer to related species such as pea^10^.

A linkage map of *L. cicera* will be crucial to identify and locate the genes and genomic regions responsible for the resistance traits, paving the way to marker assisted selection (MAS) in this orphan species. Indeed, limited genomic resources exist for this plant species, hampering its potential exploitation in legume breeding. In January 2018, the NCBI database contained only 126 sequences (considering DNA and RNA data) with almost no relevance for breeding. However, molecular tools from related species were useful in this legume. Actually, 138 molecular markers of different types previously developed for other legume species, were successfully cross-amplified in *L. cicera*^11^.

Rust fungi and particularly those from the genus *Uromyces* cause important legume diseases. *Uromyces pisi* (Pers.) Wint. causes pea rust^12^ and is also capable of infecting other species in the genera *Lathyrus*^6,13^, *Vicia* and *Lens*^14,15^.

*Lathyrus sativus* is the species most closely related to *L. cicera* and may even be derived from it^16^. These two species might, therefore, share many physiological/genetic stress-response mechanisms. We have recently characterised in more detail the response of *L. sativus* transcriptome to inoculation with *U. pisi*^17^. Differences in the reaction to infection between the resistant and susceptible genotypes investigated appeared to be mainly due to the activation of the salicylic acid (SA) pathway and expression of several pathogenesis-related (PR) genes. Indeed, in previous studies both species, *L. sativus* and *L. cicera*, showed a compatible reaction to *U. pisi*. Also, similar defence mechanisms were present, although *L. sativus* genotypes were more resistant than the *L. cicera* genotypes investigated^6,13^.

In the present work, we investigate the transcriptomes of control and rust-inoculated leaves from two *L. cicera* genotypes, with contrasting quantitative resistance to rust, to achieve several aims, such as the following: (i) to develop novel expressed simple sequence repeat (E-SSR) and single-nucleotide polymorphism (SNP)-based molecular markers for future mapping and diversity studies in *L. cicera*, (ii) to examine the differential expression of allelic variants in the two contrasting genotypes after rust inoculation and, based on the SNP information within candidate alleles, (iii) to develop appropriate assays for future quantitative trait loci (QTL)/expression QTL analysis for rust resistance in *L. cicera*. Finally, we used the developed *L. cicera* molecular markers to construct the first *L. cicera* linkage map and perform a comparative/synteny study of *L. cicera* with other legume species. This map was developed using a recombinant inbred line (RIL) population resulting from the cross of the two contrasting *L. cicera* genotypes previously analysed.

## Results

### The RNA-seq transcriptomes of *L. cicera* genotypes with contrasting resistance levels

The RNA-seq library of the susceptible genotype BGE008277 comprised 18,395,860 sequencing reads, which were assembled into 66,210 contigs, ranging in size from 150 to 8664 bp, with a mean contig length of 537 bp. The respective library from the partially resistant genotype BGE023542 comprised 30,320,831 reads, which assembled into 64,382 contigs, with a size range of 150 to 9694 bp and a mean contig length of 571 bp.

The *de* *novo* reference assembly combining the RNA-seq reads from both genotypes and from all treatments comprised 145,985 contigs, ranging in size from 150 to 13,916 bp, with a mean contig length of 485 bp. BUSCO analysis indicated that our assembly is 86% complete, showing only 4.9% of fragmented BUSCOs. In more detail, a total of 1440 BUSCO groups were found: 1238 complete BUSCOs (86%), from which 1206 single-copy BUSCOs (83.8%) and 32 duplicated BUSCOs (2.2%); fragmented BUSCOs: 70 (4.9%) and missing BUSCOs: 132 (9.1%). The mapping and quantification of both genotypes’ libraries to the reference assembly allowed the analysis of their differential expression in response to *U. pisi* infection. A total of 20,362 contigs were unique to the partially resistant and 12,114 contigs were unique to the susceptible genotype. This *de* *novo* Transcriptome Shotgun Assembly project has been deposited at DDBJ/EMBL/GenBank under the accession PRJNA264792.

### Differential gene expression in partially resistant and susceptible *L. cicera* genotypes to rust infection

*L. cicera* contigs from the susceptible and partially resistant genotype, respectively that were differentially expressed in response to rust inoculation were grouped into eight expression pattern groups (A-H) based on their up- or down-regulation (log_2_ ≥ 2 or log_2_ ≤ −2; respectively, *q* value ≤ 0.05), considering only the transcripts present in all libraries. The number of differentially expressed contigs and description of each group is summarised in Table 1. Groups with most abundant contigs were group F (contigs downregulated in both genotypes) and H (contigs downregulated only in the partially resistant genotype) with 4520 and 3498 contigs, respectively. They were followed by a group that included 2161 entries upregulated upon infection in both genotypes (group A). A detailed list including all identified contigs, their description and assignment to expression pattern groups can be found in Supplementary Table S1. As depicted in Supplementary Fig. S1, from the 111,287 contigs that could be identified and quantified, 43,590 were shared among all libraries.

**Table 1:** Classification of contigs according to their differential expression in the susceptible and resistant genotype upon infection with *U. pisi*

Expression pattern group	Feature	# of contigs
A	Upregulated in Resistant Upregulated in Susceptible	2,161
B	Upregulated in Resistant Upregulated in Susceptible, higher in Susceptible	12
C	Upregulated in Resistant, higher in Resistant Upregulated in Susceptible	20
D	Upregulated in Susceptible	1,715
E	Upregulated in Resistant	338
F	Downregulated in Resistant Downregulated in Susceptible	4,520
G	Downregulated in Susceptible	1,399
H	Downregulated in Resistant	3,498
Total		13,663

Upregulated: (log_2_ ≥ 2; *q* value ≤ 0.05); Downregulated: (log_2_ ≤ −2; *q* value ≤ 0.05); higher in Susceptible: (log_2_ fold change between all resistant and susceptible genotype contigs ≤ −2; *q* value ≤ 0.05); higher in Resistant: (log_2_ fold change between all resistant and susceptible genotype contigs ≥ 2; *q* value ≤ 0.05).

### Annotation of *L. cicera* contigs

From the 111,287 contigs detected in all libraries, 46,588 (41.9%) were matched via BLAST to entries in plant databases. A total of 622 (0.6%) contigs matched only to fungal databases and were present only in the inoculated libraries. In addition to these, 688 (0.6%) other contigs, that were also absent in non-inoculated samples, had a higher hit-score in fungal databases than in plant databases. Thus, a total of 1.2 % of all contigs most probably correspond to *U. pisi* sequences.

**Fig. 1:**
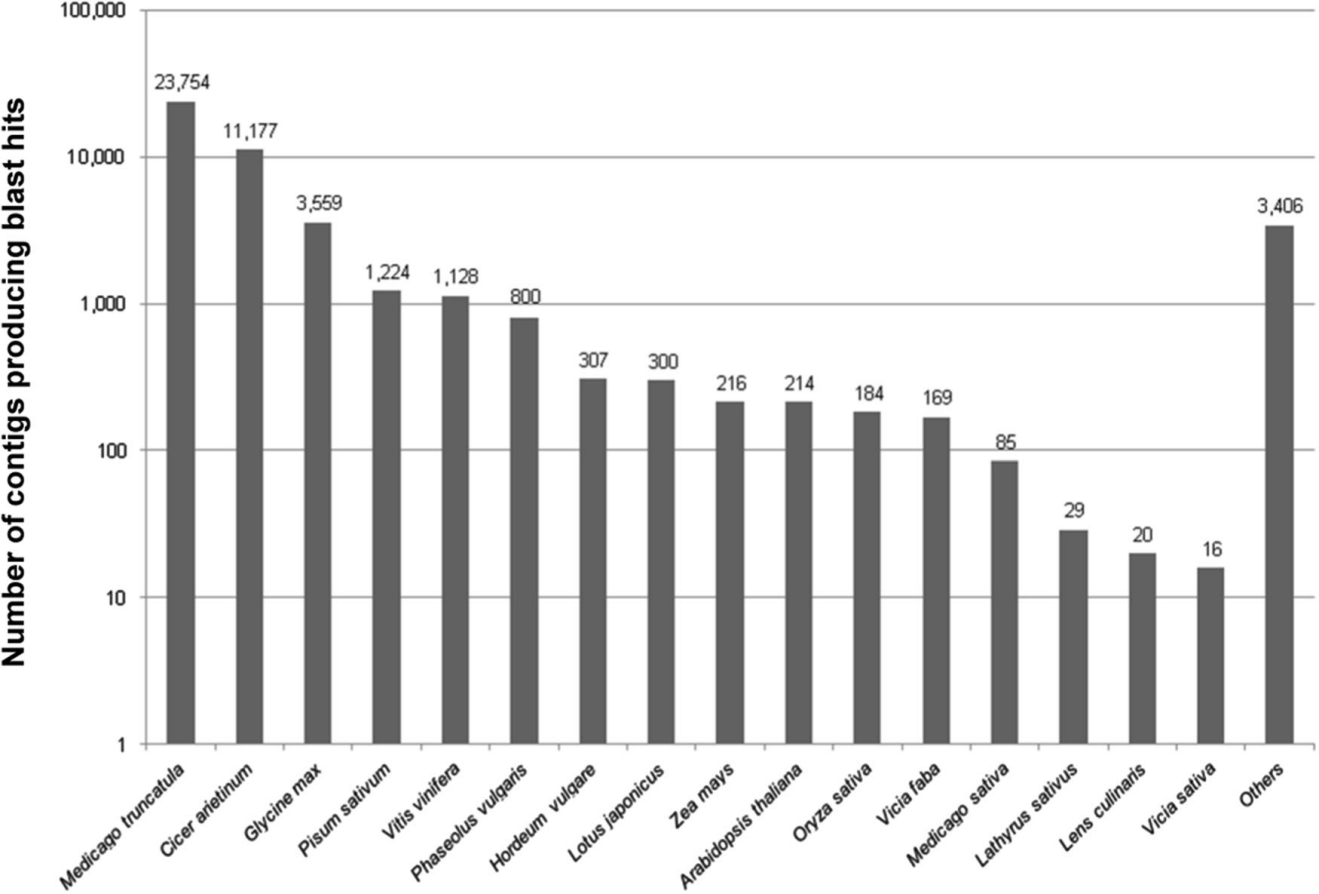
Number of contigs that could be BLASTed to different plant species

As indicated in Fig. 1, BLAST produced hits mainly to other legume species. *Medicago truncatula* (23,754; 50.99%), *Cicer arietinum* (11,177; 23.99%), *Glycine max* (3,559; 7.64%), *Pisum sativum* (1,224; 2.63%), *Phaseolus vulgaris* (800; 1.72%) and *Lotus japonicus* (300; 0.64%) were the best matching legume species. *Vitis vinifera* (1,128; 2.42%), *Hordeum vulgare* (307; 0.66%), *Zea mays* (216; 0.46%) and the model *Arabidopsis thaliana* (214; 0.46%) were the best matching non-legume species.

**Fig. 2:**
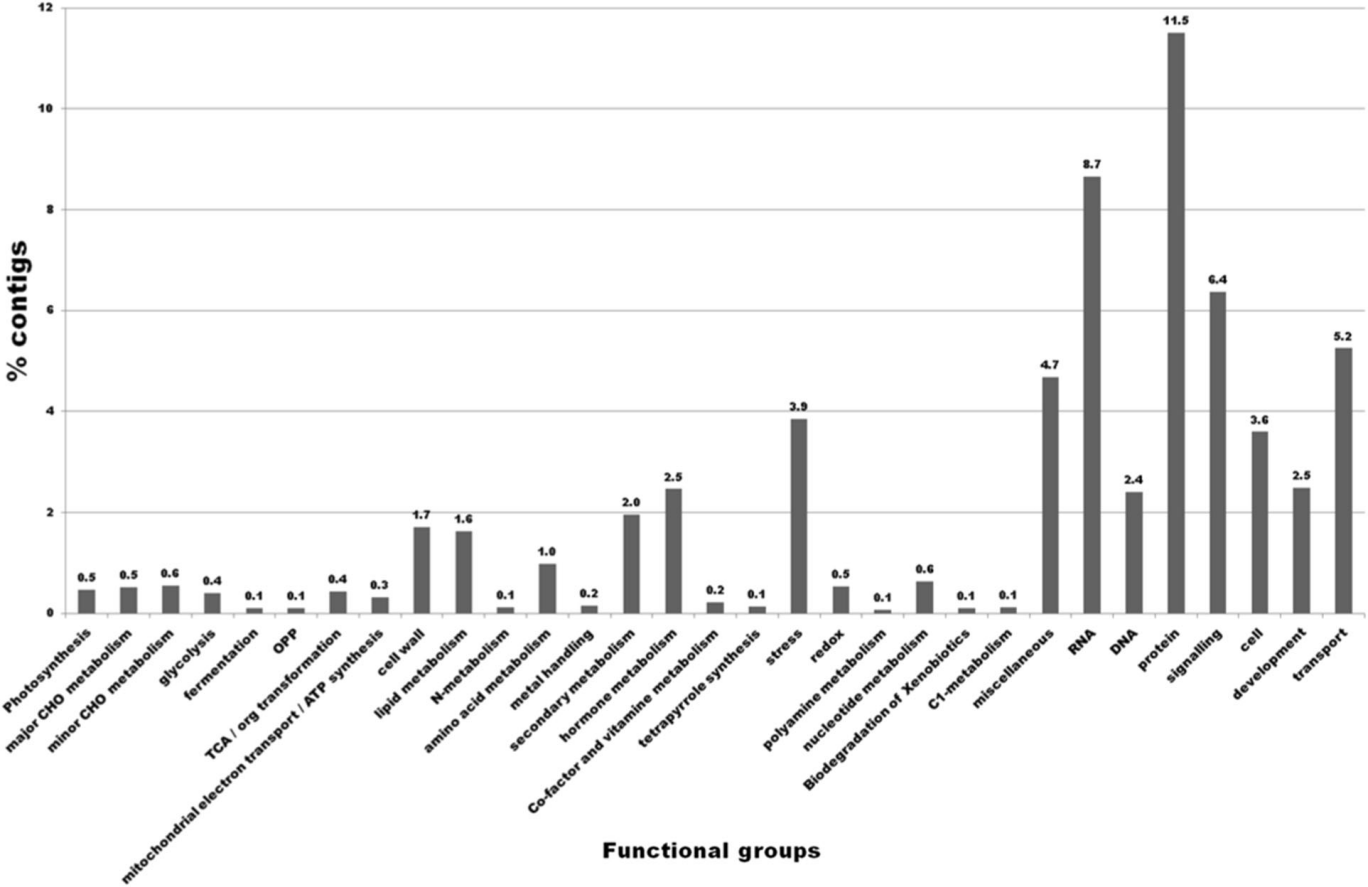
Percentage of contigs assigned to each main functional category. CHO: carbohydrate, OPP: oxidative pentose phosphate, TCA: tricarboxylic acid, ATP: adenosine triphosphate

As depicted in Fig. 2, functional annotation of all differentially expressed plant contigs via Mercator and MapMan, grouped them into 34 main functional categories, of which the categories ‘protein’ (11.5%), ‘RNA’ (8.7%), ‘signalling’ (6.4%), ‘transport’ (5.2%), ‘miscellaneous’ (4.7%), and ‘stress’ (3.9%) were most represented. A total of 37.4% differentially expressed (DE) contigs could not be assigned to any functional category.

The analysis of the identified functional categories revealed transcripts that are potentially involved in several layers of defence against pathogens^18^. Although a detailed description of these differentially expressed transcripts is not under the main scope of this study that focus on the identification of potential molecular markers useful for mapping, we emphasise those upregulated in response to rust infection only in the partially resistant genotype. This group included transcripts involved in hormone metabolism, cell wall degradation, secondary metabolism, ROS production and in signalling and regulation of transcription of defence responses. Particularly important for signalling, this group harboured ten receptor kinases, one calcium receptor and a ‘WRKY family transcription factor’. Also related to signalling, but in the group of transcripts upregulated in the susceptible and downregulated in the partially resistant genotype, a MLO-like transcript (AtMLO6, PsMLO1, a289255;11) was identified. The complete set of transcripts and its expression profiles can be found in Supplementary Table S1.

### RNA-seq validation by qRT-PCR

To validate the RNA-seq results, the expression of 8 selected genes (chosen to represent a broad range of differential expression and total transcript counts) was analysed by qRT-PCR using three biological replicates. Genes were selected according to their level of differential expression which ranged from log_2_ −1.8 to + 2.4 in the two genotypes. Primer sequences are listed in Supplementary Table S2. The normalised number of transcripts was generally higher than 100, with the exceptions of contig a20510;122, ‘Histone H2A.2’, with 28 counts in the inoculated BGE008277 and 88 counts in the inoculated BGE023542 sample, and contig a77720;50 ‘γ-tubulin’, with 73 counts in the susceptible inoculated line (Supplementary Table S3). The best reference gene assay for normalisation of qRT-PCR reactions for both genotypes, as suggested by the NormFinder software, was the transcript coding for ‘γ-tubulin’ (a77720;50). A good correlation (*R* = 0.71 for the resistant and *R* = 0.70 for the susceptible genotype) was observed between the log_2_ fold changes measured by RNA-seq and qRT-PCR.

### SNP identification in resistance pathways

A total of 19,224 Single-Nucleotide Polymorphisms (SNPs) were detected in 5,152 of the 43,590 transcripts present in both the susceptible and the partially resistant inoculated genotypes (see Supplementary Fig S1). Among them, 811 SNP-containing contigs had been functionally annotated using the MapMan software. The number of SNPs in functional categories varied considerably. Contigs in the categories ‘RNA regulation of transcription’ (6.15%) and ‘protein degradation’ (4.97%) contained by far the most SNPs, followed by the protein-related categories ‘protein postranslational modification’ (2.72%), ‘protein synthesis’ (1.89%) and ‘protein targeting’ (1.78%). Also, the categories ‘signalling receptor kinases’ (1.42%) and ‘hormone metabolism auxin’ (1.30%) contained a considerable number of contigs with SNPs (Supplementary Fig. S2). Polymorphic contigs and their respective SNPs are listed in Supplementary Table S4.

### Allele-specific expression validation by dual probe assays

Seventeen allele-specific expression assays were tested for their ability to distinguish the two alleles in the genotypes BGE023542 and BGE008277. For eight SNP sites, the allele-specific expression could be confirmed for both alleles (a22544;181, a1871;383, a42821;85, a601;548, a10029;259, a33467;117, a4980;381 and a716;325). In five cases only one of the two alleles could be unambiguously amplified (a16587;204, a12135;196, a28870;97, a1697;460 and a16062;87). For four SNP sites, neither allele could be amplified in both genotypes (a1871;383, a23248;109, a2860;344 and a19261;130) (Table 2).

**Table 2:** Allele-specific expression analysis

Reference assembly contig	SNP position	BLAST hit/[species]	BLASTn *e* value	Reference base	Variant base	BGE008277 inoculated				BGE023542 inoculated				ΔCт Mean (BGE023542 - BGE008277) reference base	ΔCт Mean (BGE023542 - BGE008277) variant base
						RNA-Seq counts reference base	RNA-Seq counts variant base	SNP assay reference base (Ct mean)	SNP assay variant base (Ct mean)	RNA-Seq counts reference base	RNA-Seq counts variant base	SNP assay reference base (Ct mean)	SNP assay variant base (Ct mean)		
a22544_181	127	Glucan endo-1,3-beta-glucosidase [*Medicago truncatula*]	3,00E-168	A	G	10	0	36.96	—	0	162	—	31.91	∞	∞
a16587_204	389	Polygalacturonase inhibiting protein [*Pisum sativum*]	0	C	T	24	0	—	—	3	186	29.91	34.97	∞	∞
a12135_196	396	Cell division protease ftsH–like protein [*Medicago truncatula*]	0	A	T	23	0	33.92	31.77	1	149	33.92	—	2.15	—
a28870_97	856	Unknown [*Medicago truncatula*]	0	G	A	1	13	35.92	—	110	0	—	—	0.40	—
a1871_383	342	Peroxidase [*Medicago truncatula*]	6,00E-150	T	C	1	19	—	—	264	0	—	—	—	—
a1871_383	593	Peroxidase [*Medicago truncatula*]	6,00E-150	C	G	35	2	30.42	29.50	0	318	30.42	29.50	−3.44	−1.55
a42821_85	1102	Calmodulin-binding heat shock protein [*Medicago truncatula*]	6,00E-150	G	A	8	0	30.01	35.19	3	38	30.72	30.96	0.71	−4.23
a601_548	419	Glucan endo-1,3-beta-d-glucosidase [*Cicer arietinum*]	0	C	G	25	164	32.17	24.17	1812	10	19.22	24.69	−12.95	0.52
a23248_109	415	ATP-dependent RNA helicase [*Medicago truncatula*]	3,00E-97	A	G	0	21	—	30.93	73	0	35.95	—	—	—
a2860_344	1236	NADP-dependent D-sorbitol-6-phosphate dehydrogenase [*Medicago truncatula*]	0	G	A	76	3	—	—	0	163	—	—	—	—
a10029_259	1404	Probable ubiquitin-conjugating enzyme E2 24-like [*Glycine max*]	0	G	A	33	0	23.94	25.71	0	101	23.35	24.99	−0.59	−0.71
a19261_130	1328	Hypothetical protein MTR_5g063940 [*Medicago truncatula*]	0	A	G	14	1	—	—	0	48	35.92	—	—	—
a33467_117	1535	Actin-related protein 2-like [*Glycine max*]	0	T	C	25	0	28.17	35.92	0	70	36.91	29.49	8.74	−6.43
a4980_381	529	Protein notum homologue [*Glycine max*]	3,00E-58	A	G	2	194	33.66	24.99	187	2	25.96	31.88	−7.70	6.89
a1697_460	388	Hypersensitive reaction associated Ca2 + -binding protein [*Medicago truncatula*]	1,00E-86	C	T	116	0	—	—	0	137	—	26.89	—	∞
a716_325	744	Ethylene-overproduction protein 1-like [*Glycine max*]	0	C	T	317	3	28.46	35.89	1	292	35.89	29.28	7.42	−6.60
a16062_87	264	Indole-3-acetic acid-amido synthetase GH3.6-like [*Glycine max*]	0	C	A	40	6	—	36.74	0	26	—	36.93	–	0.19

Summary of the qRT-PCR results in comparison to the allele distribution in RNA-seq results. Infinity - only expressed in BGE023542 or BGE008277

### Development of E-SRR markers

E-SSRs with a perfect repeat unit of two to six reiterated nucleotides were identified through the Phobos software^19^. *In toto* we developed assays for 341 expressed SSRs and tested them by PCR amplification of DNA from the two *L. cicera* genotypes. Of these, 251 produced an amplicon, where 207 (60.7%) amplicons were polymorphic between genotypes BGE008277 and BGE023542, and 44 (12.9%) were monomorphic. Thirty-one (9.1%) primer pairs produced a complex pattern and the remaining 59 (17.3%) primer pairs failed to produce any fragment. E-SSR primer pairs and genotyping results are listed in Supplementary Table S5.

### Linkage map construction

**Fig. 3:**
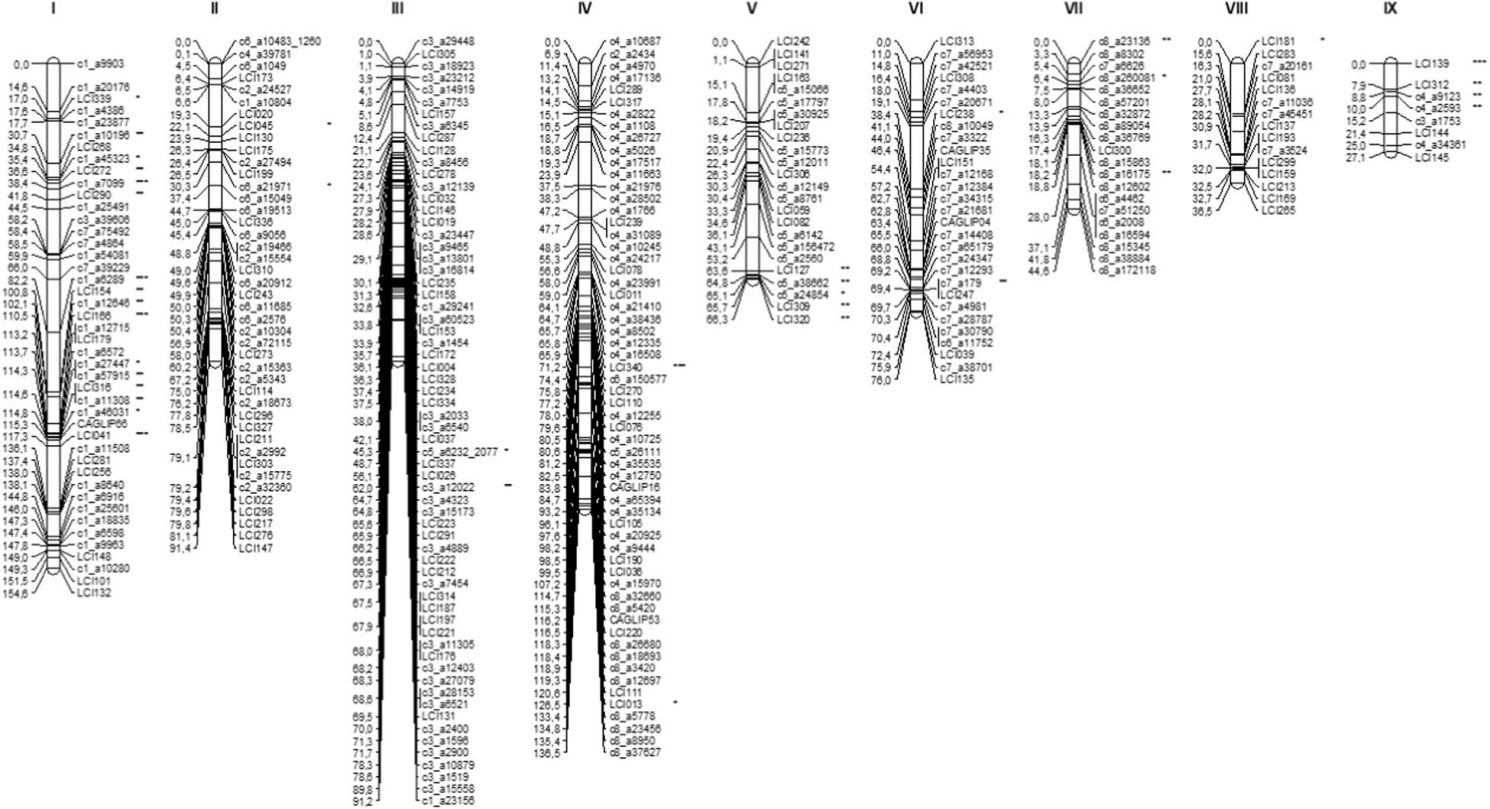
*L. cicera* first genetic linkage map based on a RIL population. Genetic distances given in cM (Kosambi mapping function) on the left. Marker names are shown on the right of each linkage group. Markers with distorted segregation ratios are marked with asterisks to their significance levels (* = 0.05, ** = 0.01 and *** = 0.005)

A total of 935 molecular markers (767 SNPs, 163 E-SSRs and 5 intron targeted amplified polymorphism (ITAP) markers from a previous study^11^, were screened in the parental genotypes of the F5 RIL population for mapping purposes. From these, 307 polymorphic loci were successfully mapped, namely, 189 SNPs, 113 E-SSRs and 5 ITAPs. In more detail, from the initial 163 E-SSRs screened in the RILs, 126 were selected for mapping, 125 segregated with the expected 1:1 Mendelian ratio (1 degree of freedom; *α* = 0.01; *χ*^2^ < 3.8) and one displayed slight segregation distortion (*χ*^2^ = 4.0). The remaining markers presented a high segregation distortion and were excluded from further analysis. From the 767 SNPs genotyped in the RIL population, 434 were withdrawn from the mapping due to several causes: 270 had more than 20% missing values, 162 were monomorphic and 2 were heterozygous in the parental genotypes. A total of 333 SNPs was then selected for developing the linkage map. Of these, 71 segregated severely distorted (*p* ≤ 0.005) and were also removed. From the remaining 262 markers, 66 were removed for having identical segregation to other markers, thus redundant for the map construction. Additionally, one RIL was removed because it had >25% of missing data. Finally, the first *L. cicera* linkage map was developed using data from 102 RILs screened with 327 polymorphic loci (126 E-SSRs, 5 ITAPs and 196 SNPs). It covered 724.2 cM of genetic distance organised in 7 major and 2 minor linkages groups (LG), with an average distance of 2.4 cM between markers. Only 5 markers could not be linked to any other LG. Eleven percent of the markers showed significant deviation from the expected 1:1 segregation ratio (segregation distortion), highlighted with asterisks in Fig. 3. A chromosomal region was considered skewed when four or more closely linked markers showed significant segregation distortion in the same direction^20^. In the present linkage map, these were observed in the extremity of LGs V and IX and in the centre of LG I. The smallest LG (IX) mainly contained markers with segregation distortion (Fig. 3).

Inspection of χ2 values for goodness-of-fit for the individual LGs gave insight into the reliability of the obtained map. The χ2 values for all the LGs were < 1, except for LG VIII (χ2 = 1.15) (Supplementary Table S6).

### Macrosynteny between *L. cicera* LGs and *M. truncatula* chromosomes

**Fig. 4:**
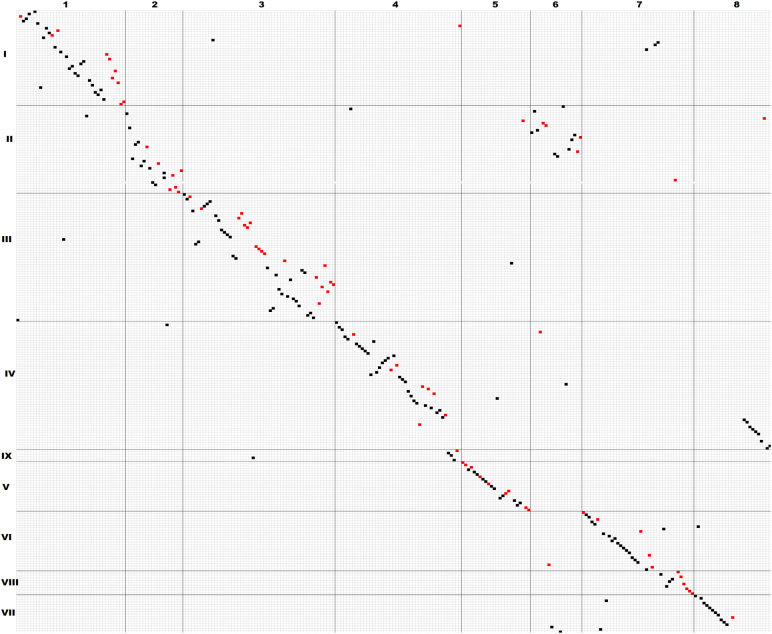
Matrix plot of common gene-based SNP and SSR markers mapped in *L. cicera* and *M. truncatula*. The *L. cicera* and *M. truncatula* loci are listed vertically and horizontally, respectively, according to their linkage group order. Black and red dots correspond to SNP and SSR markers, respectively

To obtain insight into the synteny between the *L. cicera* genome and that of other legumes we compared the order of our genic markers to the physical position of *M. truncatula* genome. Clear evidence of a simple and direct macrosynteny between the *L. cicera* and *M. truncatula* genome was detected as shown in the dot matrix in Fig. 4 and Supplementary Fig. S3. The clear isoclinic diagonal line along the linkage groups in Fig. 4 provided a strong indication of the conservation of gene order in the two legume genomes. However, chromosomal rearrangements were also evident at a moderate level. For example, *M. truncatula* chromosomes 2 and 6 merged to form the *L. cicera* LG II (Fig. 4 and Supplementary Fig. S3, C and G). Similarly, *M. truncatula* chromosome 4 splits into *L. cicera* LGs IV and IX and *M. truncatula* chromosome 7 into *L. cicera* LGs VI and VIII (Fig. 4 and Supplementary Fig. S3, E and H, respectively). Additionally, *M. truncatula* chromosome 8 spans *L. cicera* LGs VII and a large portion of the distal part of LG IV (Supplementary Fig. S3, I).

## Discussion

Previous studies on pathosystems involving *L. cicera* described its response to rust^11^, powdery mildew^7^, broomrape^9,21^ and bacterial blight^8^ at the physiological level. The present study contributes to progress in *L. cicera* research and breeding in several respects. First, it provides the sequences of more than 145,985 ESTs representing by far the most extended set of genic sequences available for this species. In addition, it detected 297 potential polymorphic E-SSRs and 19,223 SNPs in more than 5,000 transcripts and tested exemplarily whether these could be converted into molecular markers for later use in molecular breeding and their usefulness in the development of the first *L. cicera* linkage map.

The development of E-SSR markers was quite successful as it resulted in 207 novel, polymorphic marker assays, some of them we employed already here for the construction of a linkage map for *L. cicera*. Eventually these may also be used, by cross-amplification^11^, for genetic mapping in *L. sativus*. The many other genic SNPs identified here may also be converted into molecular markers. Especially the dual labelled probe qRT-PCR assays that we presented here may become useful for eQTL and allele-specific expression studies. Moreover, additional SNP arrays based on the identified SNP for diversity analysis and genetic mapping in *L. cicera* can be developed now. This was the case of the Illumina’s custom Golden Gate genotyping assay developed by Traitgenetics GmbH, Germany, that we used for genotyping the RILs mapping population in the present study, as described in Material and Methods.

The observed incongruence between the expected and experimental results in the E-SSR validation may be due to the fact that RNA-seq data provide information only from the exons. Therefore, the reason for the failure of several primer pairs to amplify a sequence at all, may be the presence of large introns between the primer binding sites which are not detectable in our RNA-seq data. Also, the production of complex patterns of fragments may be due to the existence of regions of homology to the primer binding sites elsewhere in the genome that were then amplified together with the correct fragment. Eventually, primers were located across splice sites or they could be derived from erroneously assembled contigs^22^. In any case, the fact that 82.4% of the tested SNPs could be used for the design of functional marker assays holds great promises for further molecular breeding approaches in *L. cicera*. It further demonstrates that the assemblies are correct in most cases.

The fact that only 30% of our allele-specific expression of candidate resistance gene qRT-PCR assays corroborated the RNA-seq data, points to more complex levels in the regulation of gene expression that need to be further explored^23^. We expect better results from another system with higher resolution. This will eventually increase especially the detection of rare alleles like ‘a16062;87’ with a maximum of only 40 normalised reads in RNA-seq data (Table 2).

In previous studies, resistance mechanisms against rust appeared to be similar in incompatible *L. sativus* and *L. cicera* genotypes and were consistent with a nonhost resistance response^24^. In both, *L. cicera* and *L. sativus*, resistant genotypes restrict the formation of haustoria resulting in a high percentage of early aborted fungal colonies, a decreased number of haustoria per colony and reduced intercellular growth of infection hyphae compared to susceptible genotypes from the same species^6,11,13^. Still, *L. sativus* is generally more resistant than *L. cicera*. For example, the most resistant *L. cicera* genotype (DS = 36%) found in an Iberian germplasm collection^6^, is slightly more susceptible than the most susceptible *L. sativus* genotype (DS = 30%) in the same Iberian *Lathyrus* germplasm^13^. Future comparative studies should reveal if the same defence-related genes or QTLs are involved in *L. cicera* and *L. sativus* resistance to rust, and confirm if the similarities now detected on the phenotypic resistance components would stand at molecular level. As an example, in *L. cicera* a MLO-like transcript (AtMLO6, PsMLO1, a289255;11) was upregulated in the susceptible and downregulated in the partially resistant genotype (Supplementary Table S1). The MLO gene was first identified in barley, where loss-of-function mutations in this gene conferred resistance to powdery mildew^25^. MLO-like genes mediated the vulnerability to several fungal pathogens in *Arabidopsis*^26^ and to powdery mildew in *Pisum sativum*^27^. In our previous study on rust resistance in *L. sativus*, a similar transcript was upregulated in the partially resistant genotype whereas in the resistant genotype this transcript was completely absent^17^. These findings suggest that AtMLO6/PsMLO1 homologs contribute to susceptibility in several *Lathyrus* spp., a fact that warrants an in-depth exploration in the future.

The lack of genomic resources is a common restraint to molecular analysis in orphan crops, and *L. cicera* was not an exception. From all the contigs assembled in this study, only 42% were successfully BLASTed to entries in plant databases. Since we sequenced samples inoculated with rust, we could also identify transcripts that probably have a fungal origin. Those were 1.2% of the total transcripts. As already discussed by Hacquard *et al.* ^28^, the low number of fungal transcripts may reflect the low number of fungal structures in early-infected leaves.

We developed the first linkage map for *L. cicera* based on a RIL population. This linkage map comprises different types of mostly genic molecular markers, including E-SSRs, SNPs and a few ITAPs. A total of 935 molecular markers were screened in 103 individuals from a F5 RIL population segregating for rust response to infection. The obtained map covered a total of 724.2 cM, with an average density of one marker every 2.4 cM, organised in 9 linkage groups, seven longer than 40 cM and 2 shorter groups. About 11.7% of mapped markers showed distorted segregation. These were enriched in female parent alleles and were found in clusters mainly in the extremities of linkage groups. Skewed regions may include potential lethal genes^29^, which when homozygous, may produce a lethal phenotype. Therefore, RILs containing those alleles will be absent from the mapping population. Due to linkage drag these genes would then distort the segregation of the whole-genomic region^30^.

Extensive co-linearity was detected between the *L. cicera* linkage groups and the *M. truncatula* genome. Similarly, high levels of marker order conservation have also been reported between *M. truncatula* and *P. sativum*^31^ and other closely related legumes such as *M. truncatula* and *M. sativa*^32^, *L. culinaris* and *M. truncatula*^33^ and *L. culinaris* and *P. sativum*^34^. As in these comparative studies we also identified a few rearrangements in the otherwise syntenic marker order, such as inversions (LG III and IV) and translocations (LG II and IV). In a comparison between *M. sativa* and *P. sativum* syntenic groups^35^, one re-arrangement was similar to the one we observed in *L. cicera*: linkage group (LG II) from *L. cicera* was split in two chromosomes in *M. truncatula* (Chr. 2 and 6). Also, *P. sativum*’s LG IV contained *M. sativa*’s LG 2 and LG 6[35]. We therefore hypothesise that the reduction of the number of chromosomes in *M. truncatula*/*M. sativa* (n = 8) compared to *L. cicera*/*P. sativum* (n = 7) is caused by the fusion of *M. truncatula* chromosomes 2 and 6^36^. Moreover, a translocation involving chromosomes 4 and 8 of *M. truncatula* that were represented by LG IV in *L. japonicus* was reported^37^.

In conclusion, our study provides a number of significant genetic resources for *L. cicera* in particular and in legume genomics in general. For the first time in *L. cicera* genic SSRs and SNPs were detected and validated for their use as genetic markers. Moreover, the first linkage map for *L. cicera* created with their help can now be used as a tool to localise genes governing desirable traits such as resistance to rust that segregates in the RIL population used to develop the map.

## Material and methods

### Plant material

The two studied *L. cicera* genotypes, BGE008277 and BGE023542, have contrasting quantitative resistance to rust infection. Previous evaluation of resistance levels against *U. pisi* demonstrated that BGE008277 is susceptible to rust [disease severity (DS) = 80%], whereas BGE023542 is partially resistant (DS = 36%)^6^. These genotypes were kindly provided by the Plant Genetic Resources Centre (CRF-INIA), Madrid, Spain. The mapping population used for the development of the linkage map consisted of 103 F5 Recombinant Inbred Lines (RILs) derived by single seed descendent from a cross between the two previously described *L. cicera* genotypes, BGE008277 and BGE023542.

### Rust isolate and inoculation

The *U. pisi* monosporic isolate UpCo-01 from the fungal collection of the Institute for Sustainable Agriculture-CSIC (Córdoba, Spain) was used for inoculation. Fungal spores kept at −80 °C were multiplied on plants of the susceptible *P. sativum* cv. Messire before use.

Twenty-four plants each per genotype (BGE008277/BGE023542) and treatment (inoculated/control) were used. Two-week-old *L. cicera* seedlings were inoculated by dusting all plants at the same time with 2 mg of spores per plant, diluted in pure talk (1:10), with the help of a small manual dusting device, in a completely randomised experiment. Inoculated and control plants were then incubated for 24 h at 20 °C in complete darkness and 100% relative humidity, then transferred to a growth chamber and kept at 20 ± 2 °C under 14 h light (150 μmol m^−2^ s^−1^) and 10 h dark. Plant response to infection was visually assessed 15 days after inoculation.

### RNA and DNA isolation

Total RNA for RNA-seq, qRT-PCR and SNP validation, was extracted from inoculated and non-inoculated leaves, separately from each individual plant of the two contrasting genotypes. Leaves were collected 37 h after inoculation, representing a time point in which the fungal colonies are already developing in susceptible *Lathyrus* genotypes^13^. Collected samples were immediately frozen in liquid nitrogen and stored at −80 °C. RNA was isolated using the GeneJET Plant RNA Purification Mini Kit (Thermo Scientific, Vilnius, Lithuania) according to the manufacturer’s instructions. Isolated RNA was treated with Turbo DNase I (Ambion, Austin, TX, USA), and RNA quantification was carried out using the NanoDrop device (Thermo Scientific, Passau, Germany). For the E-SSR validation and linkage map development, DNA from frozen young leaves, from the two contrasting *L. cicera* genotypes and one individual of each RIL, was extracted using a modified CTAB protocol developed by Torres *et al*.^38^.

### RNA sequencing

For each of the 4 combinations (BGE008277 control and inoculated, BGE023542 control and inoculated) total RNA was extracted individually from 24 plants per genotype and treatment and pooled in equal amounts for sequencing, in order to level out potential outliers. Five RNA-seq libraries (one for each genotype/treatment and one reference assembly which included all genotypes and treatments) were generated using a proprietary protocol (GenXPro GmbH, Frankfurt, Germany). In short, for each library, mRNA was captured from 20 µg of total RNA using Oligo dT(25) beads (Dynabeads; life Technologies). The purified mRNA was randomly fragmented in a Zn^2+^ solution to obtain approximately 250 bp long RNA fragments. cDNA was synthesised by reverse transcription starting from 6(*N*) random hexamer oligonucleotides, followed by second strand synthesis. Barcoded Y-adapters were ligated to the cDNA and the library was amplified with 10 cycles of PCR. The libraries were paired-end sequenced on an Illumina Hiseq2000 machine.

### Transcriptome assembly

Raw sequence reads were passed through quality filtering, thereby also removing sequencing adapters and cDNA synthesis primers. RNA-seq libraries from control and inoculated leaves from each of the two contrasting *L. cicera* genotypes were united prior to assembly to generate a reference assembly, enabling the generation of contigs of maximum length. All high-quality reads were *de novo* assembled by the Trinity software (Version: trinityrnaseq_r2011-11-26). In order to minimise redundancy, CAP3 software^39^ was also used with overlap length cutoff of 30 bp and overlap percent identity cutoff of 75%. Redundancy was tested using the clustering algorithm UCLUST^40^, available from (http://drive5.com/usearch/manual/uclust_algo.html). A quality check for assembly completeness was assessed with BUSCO (Benchmarking Universal Single-Copy Orthologs)^41^ against embryophyta_odb9. The resulting contigs were annotated via BLASTX to publicly available plant databases (ftp://ftp.ncbi.nlm.nih.gov/blast/db/FASTA/nr.gz, nr, plants only). To identify potential fungal transcripts, an additional BLASTX to public fungal databases (http://www.ebi.ac.uk/uniprot, UniProtKB/Swiss-Prot and UniProtKB/TrEMBL) was performed.

The sequenced reads were quantified by mapping to our own *de novo* assembled contigs with novoalign (V2.07.14; http://www.novocraft.com/). RPKM (reads per kilobase per million) were calculated as the normalised transcript expression value^42^. The obtained counts were subsequently passed through DEGSeq to calculate differential gene expression (R package version 1.16.0)^43^.

### Contig annotation and data analysis

In order to classify the obtained contigs into functional categories, the Mercator pipeline for automated sequence annotation^44^, available at http://mapman.gabipd.org/web/guest/app/mercator, was used. The mapping file was created with information from the following manually curated databases: Arabidopsis TAIR proteins (release 10), SwissProt/UniProt Plant Proteins (PPAP), TIGR5 rice proteins (ORYZA), Clusters of orthologous eukaryotic genes database (KOG), Conserved domain database (CDD) and InterPro scan (IPR). The Mercator mapping file was then employed for pathway analysis by the MapMan software^18^, available at http://mapman.gabipd.org/web/guest/mapman.

Differentially expressed contigs were identified and categorised in expression pattern groups by comparing their expression in leaves of the partially resistant genotype BGE023542, control vs. inoculated, and of the susceptible genotype BGE008277, control vs. inoculated, using DEGseq^43^.

### RNA-seq validation by quantitative RT-PCR assay

To validate the RNA-seq results, expression levels of 8 selected genes were analysed by qRT-PCR. These genes were chosen to represent a broad range of differential expression and total transcript counts. 1 μg of total RNA from each of three randomly chosen plants (3 biological replicates), from the twenty-four plants used for the RNA-seq experiment pool, per genotype per treatment (inoculated/control), was reverse transcribed in technical duplicates, using the High Capacity cDNA Reverse Transcription Kit (Applied Biosystems, Foster City, CA, USA) following the manufacturer’s instructions, in a total of six samples per genotype and per treatment. qRT-PCR reactions were performed with an iQ™5 Real-Time PCR Detection System (Bio-Rad, Munich, Germany). Primers were designed using the Primer3 software^45^. Primer sequences are listed in Supplementary Table S2. For differential expression data analysis, the Genex software package (MultiD, Goteborg, Sweden) was employed. The NormFinder software^46^, also included in Genex, was used to select the reference gene, considering the expression of γ-tubulin (a77720;50), Histone H2A.2 (a20510;122) and P700 Phospholipase (a160;902).

### SNP detection

SNPs distinguishing the transcripts of the two inoculated *L. cicera* genotypes were discovered by JointSNVMix^47^, taking as input the mappings (bam files) from the transcriptome analysis (see section “RNA sequencing” and “Transcriptome assembly”). As reference, the contigs used were obtained according to the procedure described in section “Transcriptome assembly”. The output of the JointSNVMix analysis was furthermore processed by GenXPro’s in-house software to detect SNPs discriminating the variant alleles in the inoculated samples. A minimum coverage of 15 reads in each genotype and a probability of joint genotype BB_AA and AA_BB bigger than 0.98 was needed to call a SNP.

### Allele-specific expression analysis by dual labelled probe qRT-PCR assays

In order to analyse the differential expression of allelic variants, SNPs in genes of interest discriminating the genotypes BGE023542 and BGE008277 were selected, taking into account their expression patterns, for the design of allele-specific qRT-PCR assays (Supplementary Table S7). These comprised 11 transcripts that were equally expressed between the genotypes (a11871;383 transcript present two SNP sites), one transcript with higher expression in the partially resistant genotype (a22544;181) and four transcripts with higher expression in the susceptible genotype (a4980;381, a1697;460, a716;325 and a16062;87). Altogether 2 × 17 allele-specific dual labelled probe qRT-PCR assays, with SNP-specific mismatch primers, were tested.

The primers for the dual labelled probe qRT-PCR assays (Supplementary Table S7) were designed with introduced additional mismatch in the forward or reverse primer to improve allele-specific amplification^48,49^. The expected amplicons had a size of <90 bp in order to avoid background by flanking additional SNPs (see documentation of the primer design in the Supplementary Table S8). The one-step qRT-PCR reactions were performed with 40–60 ng total RNA template per reaction from BGE023542 and BGE008277 and the corresponding 5’6-Fam-3’TQ2 dual labelled probe and the different mismatch primer for the SNP alleles in separate tubes. All reactions were made in 12 µl of One Step Prime Script™ RT-PCR Kit buffer (Perfect Real Time) from TAKARA Bio Inc., Japan. The qRT-PCR regime consisted of a reverse transcription step of 5 min at 42 °C and an initial denaturation step of 10 s at 95 °C, followed by 40 cycles of 5 s at 95 °C (denaturation) and 30 s at 62 °C (annealing/elongation).

### Development of E-SSR

The *de novo* transcriptomes were *in silico* searched for perfect SSRs with a repeat unit length of two to six nucleotides employing the Phobos^19^ plug-in for Geneious software^50^. Length polymorphisms were manually identified by aligning SSR-containing contigs of one genotype against the whole library of the other genotype.

### Molecular markers screening and map construction

The DNA from each RIL individual in addition to the two parental lines was screened using a subset of the previously developed molecular markers. In this way, 163 E-SSR markers predicted *in silico* from *L. cicera* parental genotypes RNA-Seq libraries plus 767 SNPs, selected from the same libraries taking into consideration their homology with the *Medicago truncatula* genomic sequence (MT3.5) (BLASTn; *E* value < 1E–6) and their physical position in this genome to cover evenly *M. truncatula*’s chromosomic regions (http://www.medicagohapmap.org/tools/blastsearch), preventing unwanted clustering of markers, were screened. In addition, and for the linkage map construction also five heterologous ITAP markers selected from previous publications^11^, identified as polymorphic and with an 1:1 Mendelian segregation in this mapping population were used. PCR reactions and genotyping were performed as described in Almeida *et al.* ^17^, with the exception of SNP markers that were genotyped using an Illumina’s custom Golden Gate genotyping assay provided by Traitgenetics GmbH, Germany.

Linkage analysis and segregation distortion tests were performed using JoinMap 4.0 software^54^, using a binary matrix including all the genotyping data as input. Markers with a severe segregation distortion (*p* ≤ 0.005) were removed from the original molecular data set. The determination of groups of linked markers (linkage groups-LGs) was done with a LOD score of 4. Linkage map calculations were done using all pairwise recombination estimates lower than 0.40 and a LOD score higher than 1.00, applying the Kosambi mapping function^51^. The reliability of the obtained map was checked by inspecting the individual LG *χ*^2^ value.

### Comparison with *M. truncatula* genome

Using the order of the genic SNP and SSR markers in the *L. cicera* linkage map and the information of the physical position of the same markers mapped on the *M. truncatula* genomic sequence (MT4.0)^52^ (BLASTn; *E* value < 1E–6), markers were aligned in a matrix. Co-linearity was also investigated using Strudel visualisation software^53^. Columns from the matrix correspond to the *M. truncatula* genomic sequence and rows correspond to the *L. cicera* LGs rearranged in order to facilitate the visual estimation of co-linearity.

## Electronic supplementary material

List of genes in expression pattern groups mapped to the reference assembly

List of primer sequences for the qRT-PCR experiments

Log2 fold expression results for RNA-seq and qRT-PCR experiments

List of detected polymorphic SNPs between chickling pea accessions BGE008277 and BGE023542

List of detected polymorphic E-SSRs between chickling pea genotypes BGE008277 and BGE023542

Description of the obtained linkage groups

Trancript information for allele-specific qRT-PCR assays

List of primer and probe sequences for the allele-specific expression analysis assay

Venn diagram of the number of unique and shared contigs between the two genotypes and its expression

Percentage of contigs containing SNPs between the resistant and susceptible chickling pea genotypes

Comparative plot of re-arrangement and synteny relationships between *L. cicera* and *M. truncatula*
