# Supplementary material for: First genetic linkage map of Lathyrus cicera based on RNA sequencing-derived markers: Key tool for genetic mapping of disease resistance
Source: Hortic Res. 2018 Sep 1;5:45. doi: 10.1038/s41438-018-0047-9 (PMC6119197; doi:10.1038/s41438-018-0047-9)
Supplement: Supplementary file 2 — List of primer sequences for the qRT-PCR experiments [file 41438_2018_47_MOESM2_ESM.docx]

## Supplementary Table S2 - List of primer sequences for the qRT-PCR experiments.

| **Reference assembly contig** | **BLAST hit** | **Forward primer 5´-> 3 ´** | **Reverse primer 5´-> 3 ´** |
| --- | --- | --- | --- |
|  |  |  |  |
|  |  |  |  |
|  |  |  |  |
| a160_902 | photosystem I P700 apoprotein A2 | CGAAGCATCCATGGCTGAGT | AACCCACGACTTCGCCAATT |
| a12168_173 | DEAD-box ATP-dependent RNA helicase | TCGATCAGCCTCATCAAGCAC | TGTGGTTGGTACTCCTGGGA |
| a1310_251 | Chromodomain helicase DNA-binding protein | GCTAGATGCAGCTGGACCAA | GGGCTTCATTTGCTTGTCTCAAA |
| a5102_390 | O-methyltransferase | ATTTGTCCAGGCGATGCTCA | AATATTGGGTCGTGAGCTGCA |
| a2168_503 | Nodule lectin | ACCGCTGGATAAAATCTCGGC | ATCGCACAAACCTTCCAACTCA |
| a77720_50 | γ-tubulin | GCGGCCTTCTGTCAGGTAAA | GCATCGAAACTCTCCCTCCAA |
| a19532_154 | Amino acid transporter | CTCCGTTCGTTCCATCATCCA | AGCGGTGGAGAGTACGTGTA |
| a20510_122 | Histone H2A.2 | GGCCAAGAAAACCCCCAAGA | CTTACAAGAGAGGCACGGCA |
