# Supplementary material for: First genetic linkage map of Lathyrus cicera based on RNA sequencing-derived markers: Key tool for genetic mapping of disease resistance
Source: Hortic Res. 2018 Sep 1;5:45. doi: 10.1038/s41438-018-0047-9 (PMC6119197; doi:10.1038/s41438-018-0047-9)
Supplement: Supplementary file 6 — Description of the obtained linkage groups [file 41438_2018_47_MOESM6_ESM.docx]

**Supplementary Table S6- Description of the obtained linkage groups.**

| **Linkage group** | **Number**  **of loci** | **χ2 mean** | **Length**  **(cM)** | **Average distance**  **(cM)** | **Largest**  **gap (cM)** |
| --- | --- | --- | --- | --- | --- |
| I | 44 | 0.288 | 154.6 | 3.51 | 18.8 |
| II | 43 | 0.449 | 91.4 | 2.13 | 12.7 |
| III | 60 | 0.449 | 91.2 | 1.52 | 11.3 |
| IV | 60 | 0.702 | 136.5 | 2.28 | 13.6 |
| V | 24 | 0.392 | 66.3 | 2.77 | 13.9 |
| VI | 29 | 0.156 | 76.0 | 2.62 | 19.3 |
| VII | 20 | 0.263 | 44.6 | 2.23 | 9.2 |
| VIII | 15 | 1.155 | 36.5 | 2.43 | 6.7 |
| IX | 8 | 0.180 | 27.1 | 3.39 | 6.2 |
| **Total** | 307 | - | 724.2 | - | 81.1 |
| **Average** | - | 0.448 | 80.47 | 2.4 | 9.0 |
