# Supplementary material for: First genetic linkage map of Lathyrus cicera based on RNA sequencing-derived markers: Key tool for genetic mapping of disease resistance
Source: Hortic Res. 2018 Sep 1;5:45. doi: 10.1038/s41438-018-0047-9 (PMC6119197; doi:10.1038/s41438-018-0047-9)
Supplement: Supplementary file 8 — List of primer and probe sequences for the allele-specific expression analysis assay [file 41438_2018_47_MOESM8_ESM.pdf]

Supplementary Table S8 - List of primer and probe sequences for the allele-specific expression analysis assay. Lines in green: correct primer combination, since several primer options tested for SNP positions 181, 204 & 196.

Oligo ID	Sequence
a22544-181fwd	CAGTTAGAGCAAAAGGACTTCAAT
a22544-181rev	CTTTAGACGGCGGATACGAG
a22544-181fwd2	CAGTTAGAGCAAAAGGACTTCAAG
a22544-181_bfwd	CAGTTAGAGCAAAAGGACTTCAG
a22544-181_bfwd2	CAGTTAGAGCAAAAGGACTTCAGT
a16587_204fwd1	TCATGCGACACCGACAC
a16587_204rev1	AGGAAAGGGAGATTTGTGATG
a16587_204rev2	AGGAAAGGGAGATTTGTGATGT
a16587_204_brev1	AGGAAAGGGAGATTTGTGATA
a16587_204_brev2	AGGAAAGGGAGATTTGTGATAT
a12135_196fwd1	ACCAGTTTTGGGATGGAATG
a12135_196rev1	TTGTAAGTGCTGGATATTTTAGTT
a12135_196rev2	TTGTAAGTGCTGGATATTTTAGTTA
a12135_196_brev1	TTGTAAGTGCTGGATATTTTAGTA
a12135_196_brev2	TTGTAAGTGCTGGATATTTTAGTAA
a28870_97fwd	GTCGTGTTGCATGAGAAAGAAG
a28870_97rev	GAAAGATTGTATGGTCTTCACCT
a28870_97brev	GAAAGATTGTATGGTCTTCACTT
a1871_383fwd	CGGAGTTGTGTCATGTGCTG
a1871_383rev	TTCCAAGTTTTACATTCCAAGG
a1871_383brev	TTCCAAGTTTTACATTCCAACG
a1871_383-2fwd	GTGCTTCCATTCTCCGTTTATTA
a1871_383-2rev	CGGTAAAGGTTGGTGTGTCATC
a1871_383-2bfwd	GTGCTTCCATTCTCCGTTTATCA
a42821_85fwd	TGAGGGAATTGGTGGAGAA
a42821_85rev	ATGCTCAACATCGCAGCC
a42821_85brev	ATGCTCAACATCGCAGCTG
a601_548fwd	TCCCATAATATGTCCCAGCATTTCT
a601_548rev	TTCTAATGTGAAGATTGTTGTGTCTG
a601_548bfwd	TCCCATAATATGTCCCAGCATTTGT
a23248-109fwd	ATTAGAAGGAAAAGATGTGATTGGAT
a23248-109b-fwd	ATTAGAAGGAAAAGATGTGATTGGGT
a23248-109rev	GGTTCGGCTGTGGTGCTT
a2860-344fwd	TTCATCCCTCTTCACGAGTCC
a2860-344rev	ATTGTGCTGCTGATTACAAAAACT
a2860-344b-rev	ATTGTGCTGCTGATTACAAAAATA
a10029-259fwd	ATGGTGATGTGGTCTGCTCCT
a10029-259rev	TCCTTTCACATTTTCCAAATCCG

a10029-259b-rev	TCCTTTCACATTTTCCAAATCTG
a19261-130fwd	CCATGACAATACCACGGTTGA
a19261-130rev	CCCAAATTGAAGTGTTTACGTTA
a19261-130b-rev	CCCAAATTGAAGTGTTTACGTCA
a33467_117fwd	AAAATTCTCACCGGCAAAACC
a33467_117rev	ACCGAAACTGAAAGCGCCAC
a33467_117brev	ACCGAAACTGAAAGCGCCGC
A4880-381fwd	GTCAAGAGCGCGGTAACAAAAT
A4880-381b-fwd	GTCAAGAGCGCGGTAACAAAGT
A4880-381rev	TCCCTTTTCCAAATCCTCTATCC
a1697-460fwd	TTTATATCTTCATCGCTGGCGTCT
a1697-460b-fwd	ATTTATATCTTCATCGCTGGCGTTT
a1697-460rev	AAGATGTGTTCAAGGTGATGG
a716-325fwd	GGTTGTCCCACAGCAGGAACG
a716-325b-fwd	GGTTGTCCCACAGCAGGAATG
a716-325rev	CCACCAACAACAGTGCAAGAA
a16062-87fwd	GGCGCCTCCAAAACCCG
a16062-87b-fwd	GGCGCCTCCAAAACCAG
a16062-87rev	GGTGGATCTGATAACGGTGGA

**Dual labelled probes**	**Modification: 5' 6-FAM - 3' TQ2 quencher**
a22544_181-TM	CCACGGCTATCGACATGACCCTTAT
a16587_204-TM1	TCGACCTCCCTCAACCTGTCTCCATTCC
a12135_196-TM1	ACCAACCATCTCTACTACGCAGCCAAA
a28870_97-TM	CAAAAATAATAATGAAGATGAAGATGCTGCC
a1871_383-TM	TCCGTCGCAATTCTTGGAGGC
a1871_383-2-TM	TGTCAATGGTTGTGATGGGTCAATTCTA
a42821_85-TM	TACTTTTGCTGGACATTCCCTTGGATCA
a601_548-TM	CAGCACTAGCTCCAGTTCCACCTTCAGAT
a23248-109-TM	CCGGGGCTTTTGCTCTTCCTATATTGC
a2860-344-TM	AGCGCTTCTCCAACTTCGGTTTC
a10029-259-TM	TATCAGACAGGACAAATGGGAAGGGTCA
a19261-130-TM	TGAACAACTCGATCACTACTTTCTTCAACTAATTC
a33467_117-TM	ACTTCAAATATCCGGTTCCGTTGTCGC
a4880-381-TM	TTGGATGGTAGTCCACCAGCTTACCATT
a1697-460-TM	ACCCAGCCAAAGCCATATAGTTCTTCAAATC
a716-325-TM	CAGTGGCTGGTTGTTGTTCTGGAATTGT
a16062-87-TM	AATCCCCAATTCACTCCAAACCCTTTC
