# Supplementary figures and images for: First genetic linkage map of Lathyrus cicera based on RNA sequencing-derived markers: Key tool for genetic mapping of disease resistance
Source: Hortic Res. 2018 Sep 1;5:45. doi: 10.1038/s41438-018-0047-9 (PMC6119197; doi:10.1038/s41438-018-0047-9)

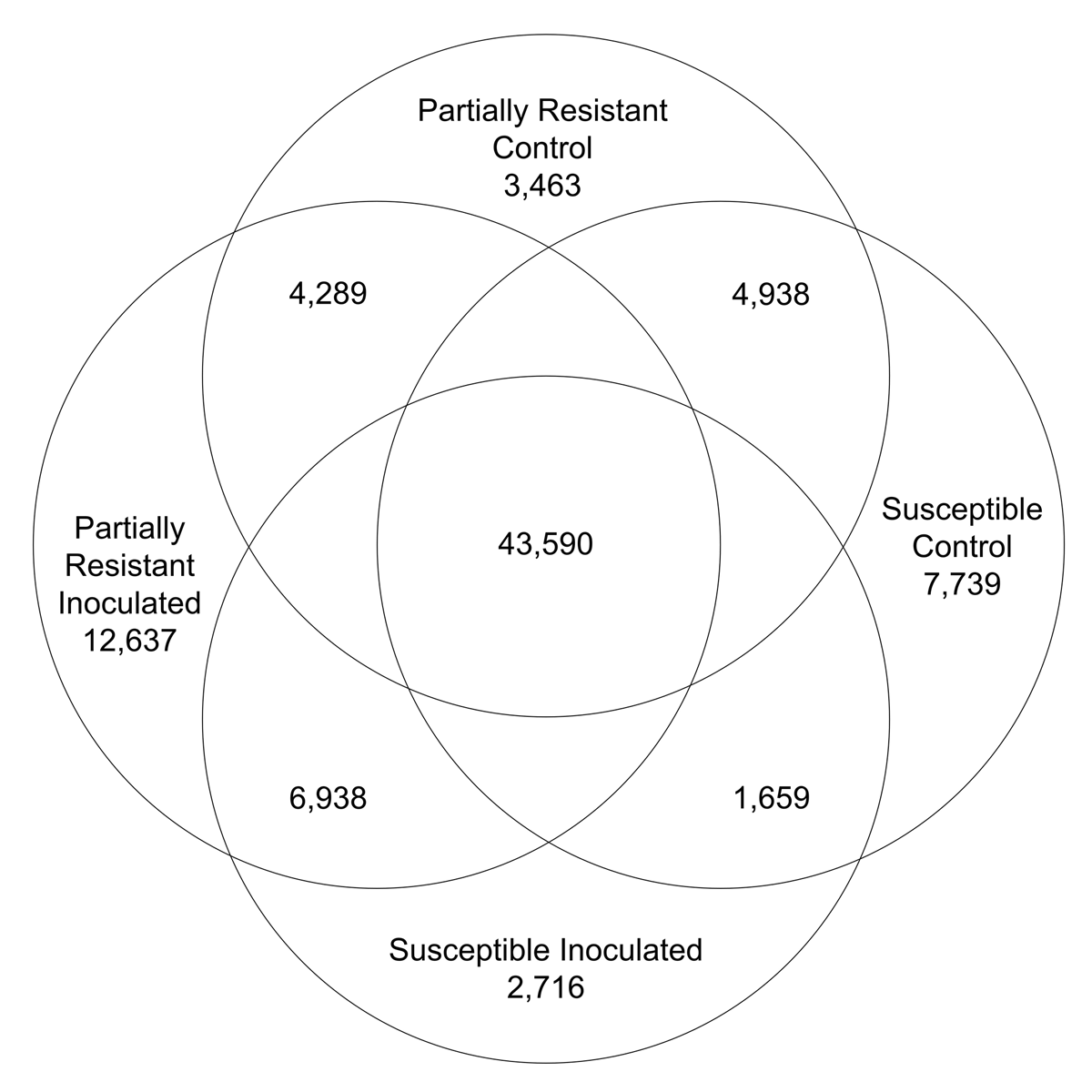

Supplement: Supplementary file 9 — Venn diagram of the number of unique and shared contigs between the two genotypes and its expression [file 41438_2018_47_MOESM9_ESM.tif]

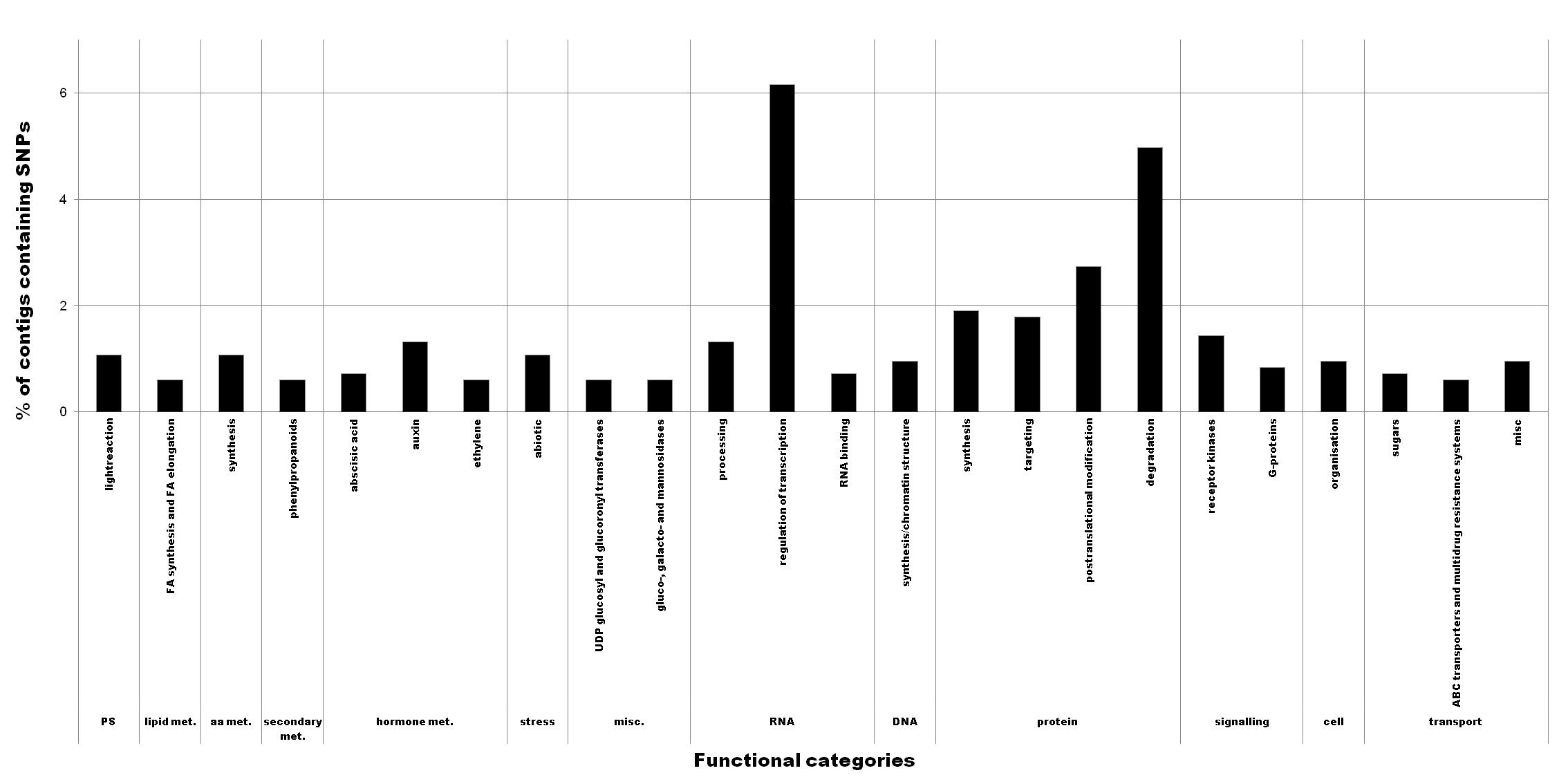

Supplement: Supplementary file 10 — Percentage of contigs containing SNPs between the resistant and susceptible chickling pea genotypes [file 41438_2018_47_MOESM10_ESM.tif]

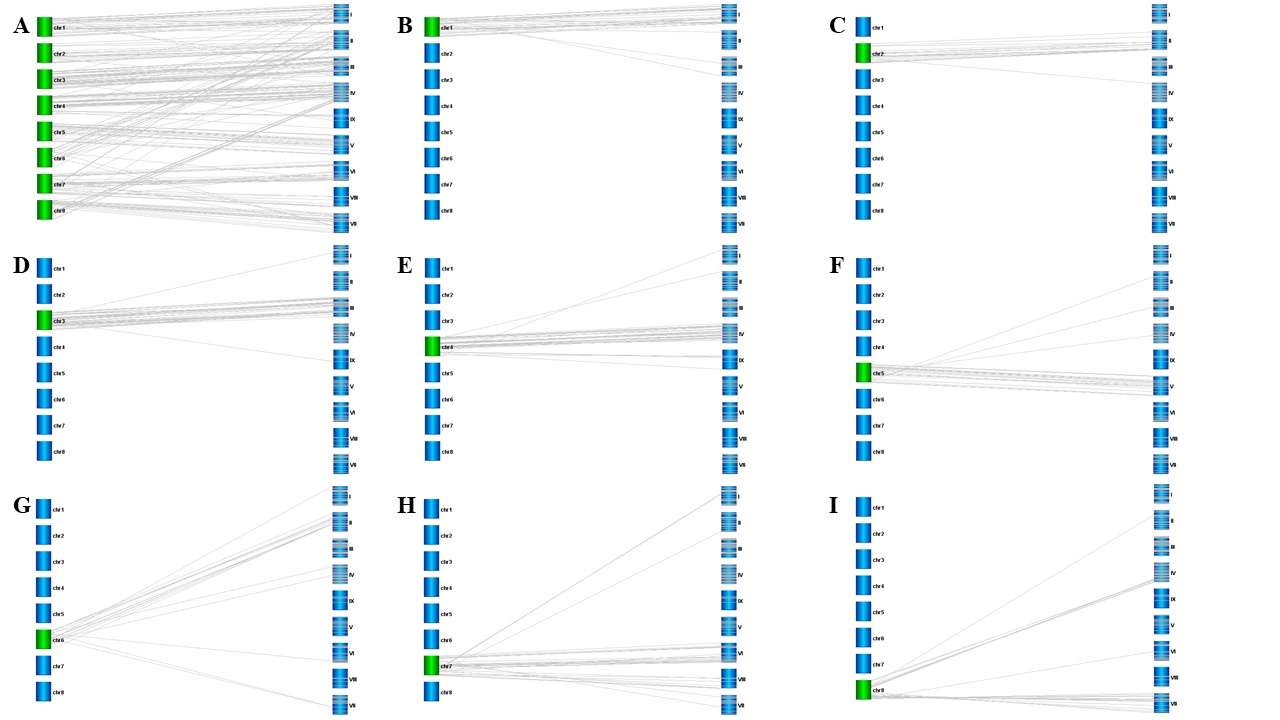

Supplement: Supplementary file 11 — Comparative plot of re-arrangement and synteny relationships between L. cicera and M. truncatula [file 41438_2018_47_MOESM11_ESM.tif]
